# Structural and electronic properties of Pt modified Au(100) surface

**DOI:** 10.1038/s41598-022-07617-2

**Published:** 2022-03-09

**Authors:** Artur Trembułowicz, Agata Sabik, Leszek Jurczyszyn

**Affiliations:** University of Wroclaw, Faculty of Physics and Astronomy, Institute of Experimental Physics, Pl. Maxa Borna 9, 50-204 Wroclaw, Poland

**Keywords:** Chemical physics, Electronic properties and materials, Phase transitions and critical phenomena, Surfaces, interfaces and thin films

## Abstract

Investigations on electronic and geometric structures of platinum adsorbed on monocrystalline gold surfaces are important for understanding the remarkable catalytic properties of bimetallic Pt–Au systems. Herein, the morphology of quasi-hexagonal (hex) Au(100) surface after deposition of platinum for coverage up to 0.5 monolayer (ML) has been investigated by scanning tunneling microscopy (STM). For coverage range 0.2–0.4 ML the creation of elongated islands with mono-atomic height is observed. The islands consist of flat phase of disordered Pt-Au alloy which coexists with nanowire-like features with a hex atom arrangement and quantized width. Annealing the Pt/Au(100) system at 100–150 °C changes the surface morphology. The islands disappear and the topmost layer of the surface consists of flat phase of Pt–Au alloy which coexists with the hex-stripes. Small domains of ordered c(2 × 2) structure of Pt–Au alloy are found. The electronic properties of this structure have been investigated by ab-initio calculations. The obtained results allow to distinguish the Pt from Au atoms by their appearance in the STM images. The calculated electronic structures indicate a bonding creation between Pt and Au atoms and an electron *d*-states redistribution of Pt in comparison to the bare Pt(100)-(1 × 1) surface.

## Introduction

Nowadays the surfaces of Pt-Au systems attract attention in the field of catalysis^1–10^. The bimetallic Au–Pt (nano)alloys usually exhibit higher catalytic selectivity and activity than either metal alone^1,4^. For instance, in the case of vicinal Au(332) surface modified by adsorption of Pt, the activation of Au atoms by alloying was found. The Au atoms were essential for dissociative adsorption of CO molecules on Pt/Au(332) surface at room temperature (RT)^9,11^. In the case of nanoalloys it was shown that the formation of Pt nanostructures on the gold based nanoparticles (NPs) improved the Pt dispersion or utilization efficiency in electrocatalysis^6,7^.

Understanding the principles underlying the reactivity and selectivity changes in alloy surfaces is critical for designing efficient catalysts. One of the factors that influences the surface catalytic properties is the electronic structure. It is already well-documented that localized *d*-states of the transition metal surfaces can significantly affect their interaction with adsorbates^8,12–15^. Strong bonding can be formed if antibonding states of adsorbate are shifted above the Fermi level and/or if bonding states are shifted below the Fermi level. Simplifying, a narrow *d*-band causes a strong chemisorption. The higher energy of the centre of the *d*-bands is, the higher in energy the antibonding states are, which results in both the strengthening of the molecule–substrate bond and the weakening of the intramolecular bond of the adsorbed molecule. For example, it was shown for Au(111) covered by Pt that the upshift of *d*-band centre is responsible for a stronger bonding of CO molecules to the mixed Pt–Au islands than to the clean Pt(111) surface^10,13^. The influence of *d*-band upshift in Pt–Au system on the enhancement of the catalytic properties has been recently observed for a platinum monolayer (ML) on gold NPs^8^. The particles exhibit remarkable selectivity in the hydrogenation of halonitrobenzene to haloaniline. One of the reasons for such high activity is the upshift of the platinum 5*d*-band centre through platinum lattice expansion^8^. The nanosize of particles made their surfaces investigation difficult and thus the impact of nanosurface electronic and geometric structure on catalytic properties is often unclear. Therefore, the studies of model systems, like ultra-thin films of platinum on gold monocrystalline surfaces under well-defined experimental conditions are important in order to better understand the processes responsible for catalytic activity of bimetallic NPs.

Gold as well as platinum crystallize in a face-centered cubic (fcc) structure with the unit cells equal a_Au_ = 4.08 Å and a_Pt_ = 3.92 Å, and thus the lattice misfit is about 4%. For both metals, their topmost layer of {100} surfaces displays the quasi-hexagonal (hex) reconstruction instead of squared lattice^16–21^. The atom density of hex structure of Au(100) or Pt(100) is about 25% higher than that for the unreconstructed (1 × 1) phase. The atom compression in the topmost layer leads to various non-equivalent adsorption sites occupation by surface atoms on the unreconstructed second layer, ranging from hollow to top sites^17,22^. The scanning tunneling microscopy (STM) investigation of hex-phases revealed the atomic rows along the <011> directions^17,18,21,22^. The various vertical atoms positions result in their elevations and diminutions along as well as across the atomic rows. The hex structure can be described with respect to the second layer by simplified (5 × 28) unit cell. The hex-reconstruction of Au(100) surface can be transformed into a (1 × 1) phase through deposition of adsorbates, e.g. platinum^23,24^ or iron^24–26^. Also a low energy ion (Ar or Ne) irradiation of bare hex-Au(100) leads to atoms rearrangement^27^. After ion exposition the coexistence of (1 × 1) phase with the hex areas, which number of atomic rows is quantized (‘magic’) and given by formula (6n + 1) (n is natural) has been found^27^. Above formula also describes the width of the energetically favorable configurations of Au islands on the hex-Au(100) surface studied by molecular dynamics (MD) simulations^28^.

The dehydrogenation rate subsequently increases with the platinum coverage up to 1.5 ML and then reaches maximum, which was about sixfold relatively to clean Pt(100), between 1.5 and 2 ML. The direct explanation for improvement of catalytic Pt properties was not determined, however the possible reasons of activity enhancement, such as the Pt edge atoms in crystallites that are more active than atoms in a smooth plane or electronic interactions between platinum and gold which might affect the bonding of hydrocarbons were pointed out^29,30^. The latter were characterized by photoemission investigations, where formation of interface state at binding energy (BE) of 1.0 eV for 1 ML of Pt was found^31^. The feature is not observed for spectra obtained for clean Au(100) or for thicker films of Pt^31^. Better insight into morphology of Pt/Au(100) system (for coverage up to 0.5 ML) has recently been obtained by STM investigations^24^. At coverage range from 0.05 to 0.4 ML the presence of anisotropic, rectangular/elongated, monoatomic Pt islands of which the long axis is parallel to rows of the hex-Au(100) surface was reported. Subsequent increasing the amount of Pt from 0.15 to 0.4 ML resulted in locally lifting the Au(100) reconstruction around the ad-metal islands with a critical size about 10 nm. In consistency with previous LEED investigations, the reconstruction disappeared at coverage of 0.5 ML. Due to Pt coverage determined from X-ray photoelectron spectroscopy (XPS) being lower than the one obtained from STM, the possibility of Pt–Au alloy formation has been suggested^24^.

The early studies on structural properties of Pt on Au(100) were performed by low-energy electron diffraction (LEED) and Auger electron spectroscopy (AES) methods^23^. Deposition of platinum at room temperature (RT) transforms the hex reconstruction to (1 × 1) structure at a coverage of about 0.5 ML. The diffraction pattern remains unchanged up to 8 ML, i.e. the highest studied coverage, as well as during the sample annealing at temperatures range 425–475 K. The occurrence of diffusion of Pt into the gold crystal after annealing at 520 K was detected^23^. The AES results indicated the Volmer–Weber growth mode, i.e. the formation of 3D crystallites of Pt on gold surface. Apart from the determination of structural properties, the authors shown that the Pt/Au(100) (up to 4 ML) system exhibits a remarkable activity for cyclohexene dehydrogenation to benzene^29,30^.

Since the exact morphology of islands observed after deposition of sub-monolayers of Pt on Au(100) as well as their thermal stability are still open issues, we performed the atomic scaled STM investigations and density functional theory (DFT) calculations on the Pt/Au(100) system. The presence of Pt-Au alloy on the sample surface before and after annealing is found. The alloy is mainly disordered, however a c(2 × 2) structure is locally detected by STM after annealing. The DFT studies on this structure revealed an electron states redistribution for Pt atoms in comparison to those in smooth Pt(100)-(1 × 1) surface, which is in agreement with previous photoemission studies.

## Methodology

The measurements were performed in situ in an ultra-high vacuum (UHV) chamber with the base pressure below 2·10^–10^ Torr by means of Aarhus STM. STM topographies were recorded at room temperature (RT) in the constant current mode with a tungsten tip cleaned by an argon ion beam etching. The STM images were prepared and analyzed using WS × M software from Nanotec^32^, where the following filters were used: Reverse Fast Fourier Transform, Remove Lines, and Smooth. An Au(100) single crystal was cleaned by cycles of Ar ion sputtering with the energy of 3 keV for 60 min; this was followed by 5 min annealing up to 900 K. The sample was annealed using electron bombardment from a tungsten filament mounted at the backside of the sample. The sample temperature was controlled by measuring sample current and calibrated on a tantalum plate with spot welded thermocouple type K. The cleanliness of the sample was checked by the STM at RT. Platinum was deposited from Pt 99.95% wire wound on a W filament heated by electron bombardment. The deposition rate was 0.05 ML/min and was established (based on series of STM images) by determination of the surface area covered by rectangular hex-reconstructed islands visible after Pt deposition.

Theoretical part of our study was based on density functional theory (DFT) as it is implemented in VASP package^33–36^. In the performed calculations the plane wave basis set was applied while the electron–ion interactions were simulated with the use of PAW^37,38^ potentials. The exchange-correlations effects were described in the framework of generalized gradient approximation (GGA) in its PBE formulation^39,40^. The energy convergence of the electronic states was controlled with the help of Davison-Block algorithm^41^.

The description of Au(100) substrate has been performed with the use of an asymmetric slab composed of seven atomic layers with about 25 Å vacuum region. The bulk lattice constant obtained from minimum energy condition of the bulk unit cell equals 4.06 Å and was fully optimized. The surface unit cell size used for the c(2 × 2) structure calculation was the elementary unit cell of the fcc(100) structure and consisted of 1 Pt and 1 Au atom. The whole slab contained 14 atoms. During the relaxation procedure the atomic positions of the atoms from the five topmost layers were allowed to relax until the forces were reduced up to the level below 0.0001 eV/Å, while the atoms from the rest part of the system were frozen in their bulk-like positions. The irreducible part of the corresponding surface Brillouin-zone was sampled by the net of 110 k-points, however, the influence of this factor on the obtained results was tested in separate check calculations.

To improve the comparison of the structural and electronic properties of considered system obtained from our theoretical study with corresponding experimental data provided by STM measurements we have performed the simulations of the STM images based on the Tersoff–Hamann approach^42^, as it is implemented in Hive code^43^. This code uses the partial charge distributions obtained from VASP calculations and enables us to simulate, in the constant current mode, the STM topographies and profiles which could be directly compared with corresponding experimental data. The analysis and visualization of DFT results has been also performed in VESTA^44^ and p4vasp^45^ programs.

## Results and discussion

**Figure 1:**
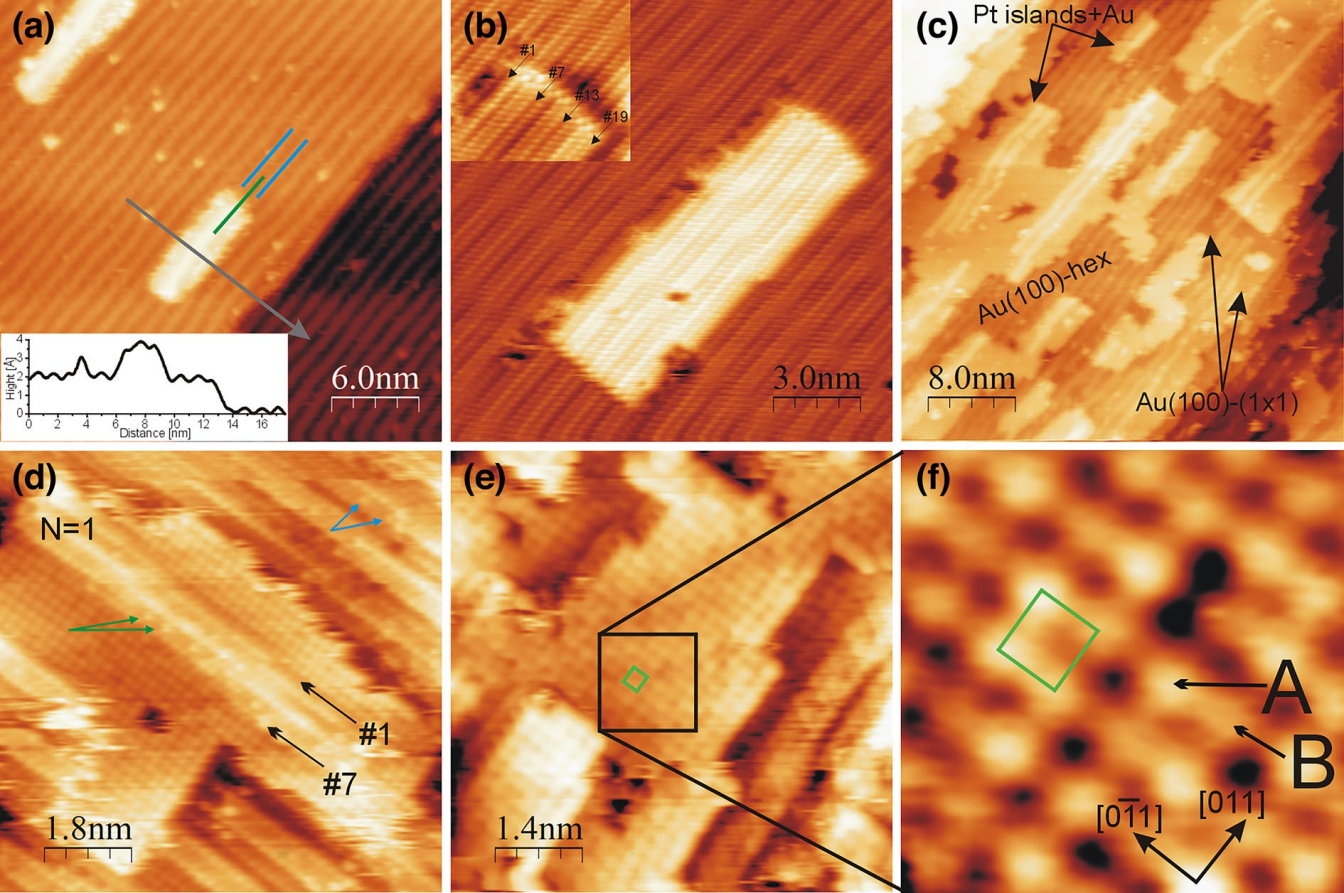
STM images of Au(100) after deposition of: **(a)** 0.05 ML Pt; **(b)** layer from **(a)** after subsequent annealing at 50, 80 and 100 °C for 10 min; **(c,d)** 0.3 ML; **(e,f)** 0.5 ML Pt. Taken for: **(a)** U =  + 758 mV; I = 2.1 nA; **(b)** U =  + 9 mV; I = 5.4 nA; **(c)** U = − 464 mV; I = 4.5 nA; **(d)** U = − 3 mV; I = 5.3 nA; **(e,f)** U =  + 3 mV; I = 4.7 nA.

In this work platinum is deposited onto the hex-reconstructed Au(100) surface at RT. For low coverages, up to about 0.15 ML, the formation of small rectangular islands on the substrate is observed by STM. The representative STM image of surface terraces after deposition of about 0.05 ML Pt is presented in Fig. 1a. The hex-stripes are clearly visible on the uncovered areas and indicate that the surface is hex-reconstructed there. The long axis of islands is parallel to the atomic rows of reconstructed substrate. The apparent height of island relative to the hex-Au(100) surface, see the line profile along the arrow in Fig. 1a, is about 2.0 Å. This value is consistent with the apparent height of step edge and indicates that the islands have monoatomic height. The islands are present on the surface even after subsequent sample annealing at 50 °C, 80 °C, 100 °C for 10 min. The STM images with atomic resolution, see Fig. 1b as an example, confirm the substrate hex-reconstruction in uncovered areas, and reveal the arrangement of atoms at the islands. The structural similarities between the atoms creating the island and those in the substrate surface are observed. The islands consist of atoms arranged in the quasi-hexagonal structure. Moreover, the number of atomic rows building the islands is quantized and follows the formula (6n + 1), which was reported previously for describing the ‘magic widths’ of Au islands on hex-Au(100)^28^ and Au nanowire-like features^27^. The island shown in Fig. 1b contains 19 atomic rows, i.e. n = 3, see the insert. The hex stripes of the island are not a continuation of the substrate pattern—the corresponding valleys and ridges are not aligned. The mismatch is also present before annealing at RT—see Fig. 1a, where the hex-stripes on the substrate and the island marked by blue and green arrow, respectively, are shifted. Surface morphology observed by us is consistent with the previous STM investigations on the Pt/Au(100) system reported in Ref.^24^ Therein, the formation of rectangular islands aligned with the hex-stripes for coverages 0.05–0.15 ML at RT was found. The islands were described to be built of platinum through which the imaging of Au surface row corrugation occurred^24^. Comparable observation was made in the same work for the islands obtained after deposition of about 0.1 ML of iron and chromium. On the other hand in the works^25,26^ on the Fe/Au(100) system, it was shown by scanning tunneling spectroscopy, that the rectangular islands with the hexagonal atom arrangement are built of gold. The formation of gold islands was explained by the exchange process of Fe atoms at RT with the atoms in the hex-Au surface. The gold atoms expelled from the substrate create the islands^25,26^. In the case of our study we could not determine the composition of the islands unambiguously. However, the mismatch of hex-patterns between the islands and substrate observed in the images with atomic resolution indicates the hex atom arrangement at the islands rather than scanning through the ad-layer. Moreover the shape and ‘magic’ width of islands are in agreement with those previously found for Au overlayers on the hex-reconstructed Au(100)^19,28^. The rectangular islands with the hex atom arrangement were detected by STM during homoepitaxy on Au(100)^19^, whereas the MD simulations revealed that widths of (6n + 1) rows are the energetically favorable configurations for Au islands^28^.

Increase the Pt coverage by subsequent deposition leads to lateral growth of islands on the surface. In Fig. 1c the STM images obtained for 0.3 ML Pt on the hex-Au(100) surface are presented. Herein, the islands are not perfectly rectangular but still have elongated shape. Behind the islands the substrate morphology could be determined. The uncovered substrate topmost layer mostly consists of the hex-stripes (assigned as Au(100)-hex in Fig. 1c), which indicates the hex- reconstruction of surface. Again the long axis of islands is parallel to the substrate stripes. Those stripes coexist with the small flat areas, located mainly beside the shorter edges of islands. Exemplary flat areas are labeled as (1 × 1) in Fig. 1c. The magnification of this phase reveals that it is the (1 × 1) structure. The presence of flat phase implies that the growth of islands is accompanied by partially lifting the hex reconstruction of Au(100). The islands morphology is also not uniform and two structures could be distinguished i.e. flat phase and nanowire-like features. In contrast to the substrate morphology, herein the flat phase prevails under the nanowire-like structures. The apparent height difference between those two phases is 0.6 Å. The value is in good agreement with the previously reported apparent height difference between the (1 × 1) and hex phases, which coexist in the topmost layer of Au(100)^27^. Therefore, we conclude that those two phases creates single ad-layer rather than there is a growth of 3D islands with flat first ad-layer and the nanowire-like features on it as the second ad-layer. The nanowires-like structures on the islands are aligned with those observed on the substrate.

We determine the islands composition from the STM images with atomic resolution, representatives are shown in Fig. 1d–f. In Fig. 1d the coexistence of flat phase with nanowire-like feature at single island is visible. The (1 × 1) structure for flat areas is found. The nanowire-like structure consists of atoms arranged in the rows which exhibit STM contrast modulation comparable to one recorded for hex-stripes of Au(100) reconstruction. However, there are some atoms appearing brighter than expected from their positions within the row. Examples of such atoms are assigned in Fig. 1d by green arrows. The apparent height difference between bright atom and the neighboring atoms in the same rows is in the range of 0.1 Å. Therefore, the bright species are not adatoms, but belong to the hex-row. It is worth to emphasize that comparable brighter species appear in the hex-stripes at the uncovered substrate surface, which is indicated by blue arrows. As it will be shown later, the bright ones are Pt and the dark ones Au atoms. This reinforces our supposition that the growth mechanism is similar to that observed for Fe/Au(100)^25,26^. The nanowire-like structures at the islands exhibit the ‘magic widths’. For instance the ones in Fig. 1d consists of seven atomic rows (the utmost ones are labeled as #1 and #7), which is in accord with the formula on ‘magic width’ for n = 1. The flat phase at the islands appears with non-uniform STM contrast, i.e. bright and dark areas are distinguishable e.g. in Fig. 1d as well as in Fig. 1e where the island obtained after deposition of 0.5 ML of Pt is visible. The provided in Fig. 1f high-resolution STM image of area marked by black square in Fig. 1e clearly reveals the (1 × 1) structure. Its unit cell is marked by blue square. The phase consists of two kinds of atoms bright (labeled as A) and dark (marked as B) with the apparent height difference of about 0.1 Å. This value is comparable to one obtained for bright atoms and their neighbors in the nanowire-like feature (in Fig. 1d). The differences in the STM appearance of atoms we attribute to different chemical contrast^46^ between platinum and gold. Thus, the (1 × 1) structure is composed of disordered Pt-Au alloy and there are some exchanges of atoms in the nanowire-like structures. Since we expect that hex-stripes at the surface mostly consist on gold atoms, we ascribe Pt atoms to the brighter features visible in the STM image presented in Fig. 1d. The possibility of Pt-Au alloy formation for Pt/Au(100) system was previously suggested in Ref.^24^, where the coverage determined from the size of platinum islands observed in the STM images was higher than the one obtained from the XPS analysis. Moreover, the presence of bimetallic islands of Pt–Au alloy has been found after deposition of about 0.04 ML of Au on the hex-Pt(100) surface by STM^47^. Therein the atoms in the islands were arranged in the square (1 × 1) structure and the islands contain some Pt atoms that, depending of the imaging conditions, appeared as darker or brighter features.

**Figure 2:**
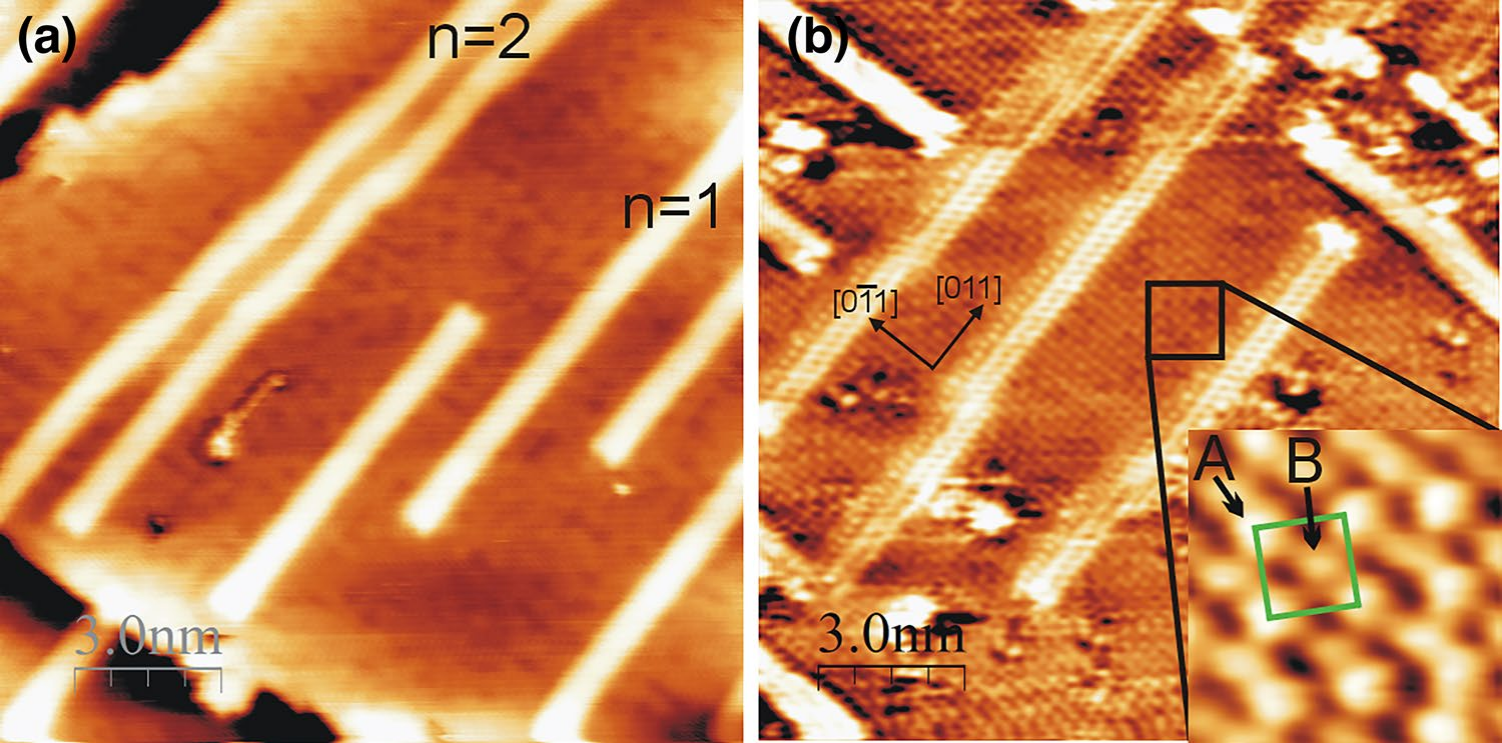
STM images of Pt/Au(100) obtained after annealing of **(a)** 0.3 ML at 100 °C; **(b)** 0.4 ML at 100 ÷ 200 °C. Taken for **(a)** U = − 1489 mV; I = 8.1 nA; **(b)** U =  + 215 mV; I = 8.7 nA; insert: U = −132 mV; I = 8.9 nA.

Annealing the Pt/Au(100) systems (for 0.2–0.4 ML) between 100 and 150 °C changes the surface morphology. In Fig. 2a the STM image obtained after annealing at ~ 100 °C the 0.3 ML of Pt on Au(100) is shown. The presented surface state is typical for post-annealing Pt/Au(100) systems for coverage range between 0.2 and 0.4 ML and temperatures higher than 100 °C. The islands are not present on the surface anymore. Now, only the topmost layer of the sample is detected, which consists of nanowire-like features coexisting with the flat areas. They have the ‘magic widths’, for instance in Fig. 2a five nanowire-like structures are visible—their widths are in accord with the formula on the ‘magic widths’ for n = 1 (four of them) and n = 2 (the one on the left side of image). The flat phase morphology is comparable to the one observed at the islands before annealing, i.e. dark and bright areas are distinguishable. The high resolution STM images, as presented in Fig. 2b, show that atoms are arranged in the (1 × 1) structure. Moreover, the phase contains bright (A) and dark (B) atoms with the apparent height difference between them of about 0.1 Å. Therefore, the flat phase, similar to the unheated layer, is composed of Pt-Au mixture. The majority of the alloy is disordered, however small compositionally ordered domains of the c(2 × 2) structure are observed, see the insert in Fig. 2b. The unit cell of the c(2 × 2) structure is drawn by green square. The structural properties up to 8 ML of Pt on Au(100) after annealing at 425–475 K was investigated by LEED^23^. Therein only the (1 × 1) structure was detected before and after sample annealing. However, the diffusion of Pt into the gold crystal after annealing at 520 K was found by AES^23^. Lack of LEED pattern associated with the formation of the c(2 × 2) structure may arise from the very small size of compositionally ordered domains and thus unavailability to detect them by LEED. The diffraction method needs at least an order arrangement of atoms in a size larger than the coherence length of the probing electrons, which differs from different instruments but is in the order of a hundred of Ångstroms.

**Figure 3:**
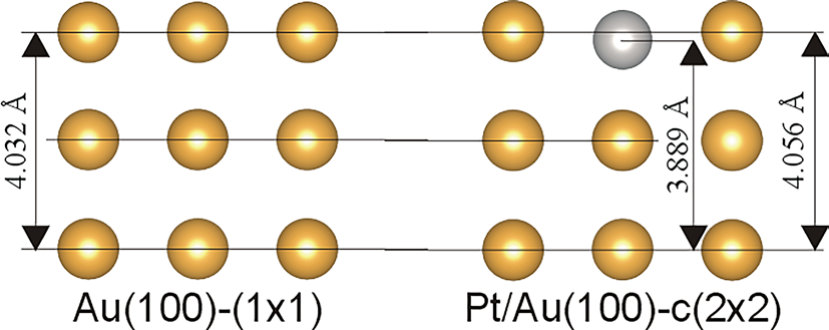
Geometry properties of the relaxed Au(100)-(1 × 1) and Pt/Au(100)-c(2 × 2) structures.

The described above STM data reveal that annealing of Pt/Au(100) system leads to development of small ordered domains of Pt-Au surface alloy. Two kinds of atoms, arranged in the c(2 × 2) structure, are clearly distinguishable in the STM images, however their exact identification is not possible based on the presented experimental results. In order to ascribe an atom (Pt or Au) to its STM appearance, we have performed DFT calculations. Two surface structures, i.e. the (1 × 1) of Au(100) and the c(2 × 2) of Pt-Au mixture on Au(100)-(1 × 1), have been considered. The results of multilayer relaxation of both structures are summarized in Fig. 3. The relaxation process moves Pt about 0.16 Å below the topmost gold atoms. The influence of Pt on the vertical position of topmost gold atoms is negligible, i.e. they are shifted upwards about 0.02 Å in comparison to those in the Au(100)-(1 × 1) structure. The diminutions of vertical position of Pt atoms in comparison to gold atoms in the first layer is not surprising, since Pt is a smaller atom than Au.

**Figure 4:**
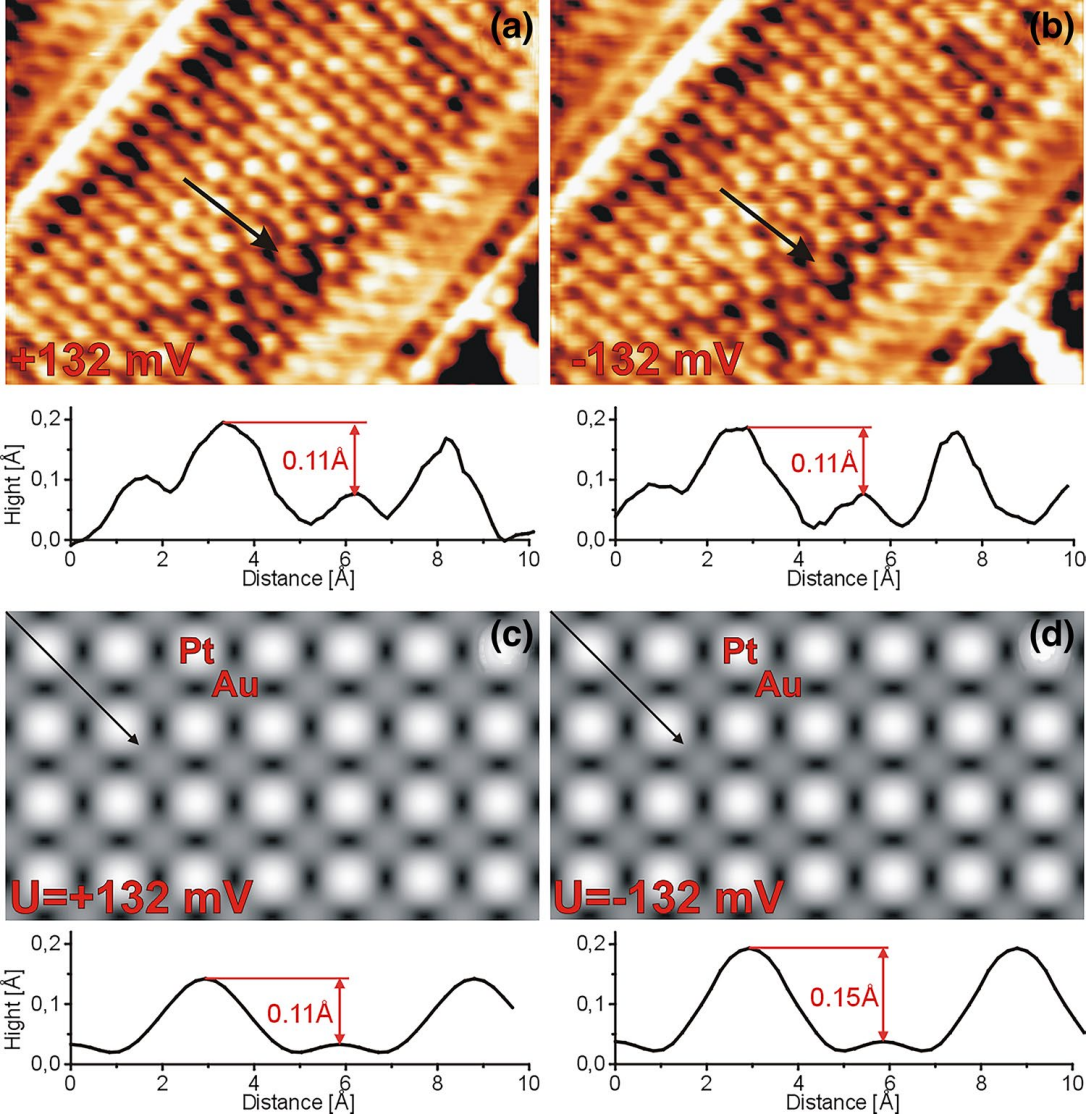
Comparison of STM images of c(2 × 2) structure taken for **(a)** U =  + 132 mV; I = 8.7 nA and **(b)** U = −132 mV; I = 8.7 nA with the calculated images for **(c)** U =  + 132 mV and **(d)** U = − 132 mV.

The standard way in the interpretation of the topographies of the images provided by STM measurements is based on the assumption that they reproduce the distribution of these electronic states at the sample surface which are active in tunneling process. To compare the obtained STM data with corresponding surface electronic structure obtained from our theoretical study we have performed the simulation of STM images. These simulations were based on the calculated spatial charge distributions of the electronic states from the following energy ranges: from −0.132 eV up to the Fermi level (occupied states) and from the Fermi level up to + 0.132 eV (unoccupied states). These two energy windows correspond with bias voltages 0.132 V, which were used in our measurements shown in Fig. 4. This figure presents the comparison between STM images obtained for occupied and unoccupied states (a), and (b), respectively (bias −0.132 and + 0.132 V) and corresponding simulated STM images calculated for the same energy ranges (i.e. for occupied and unoccupied states shown in (c) and (d), respectively). The presented images were prepared for the charge density level equal to 14 × 10^–6^ e/Å^3^, which correspond to the images obtained for the tip hold 3.1 Å above the highest atom. Moreover, Fig. 4a,b also present STM profiles from STM images. Corresponding dependencies extracted from the calculated images are also presented in Fig. 4c,d. The comparison of the experimental data with the corresponding STM simulations shown in Fig. 4 indicate the good agreement between experimental and theoretical results. Calculated STM images presented in Fig. 4c,d reproduce the topographies of STM images provided by our measurements shown in Fig. 4a,b very well. Also the STM profiles extracted from experimental data are well restored by the corresponding profiles obtained from STM simulations.

**Figure 5:**
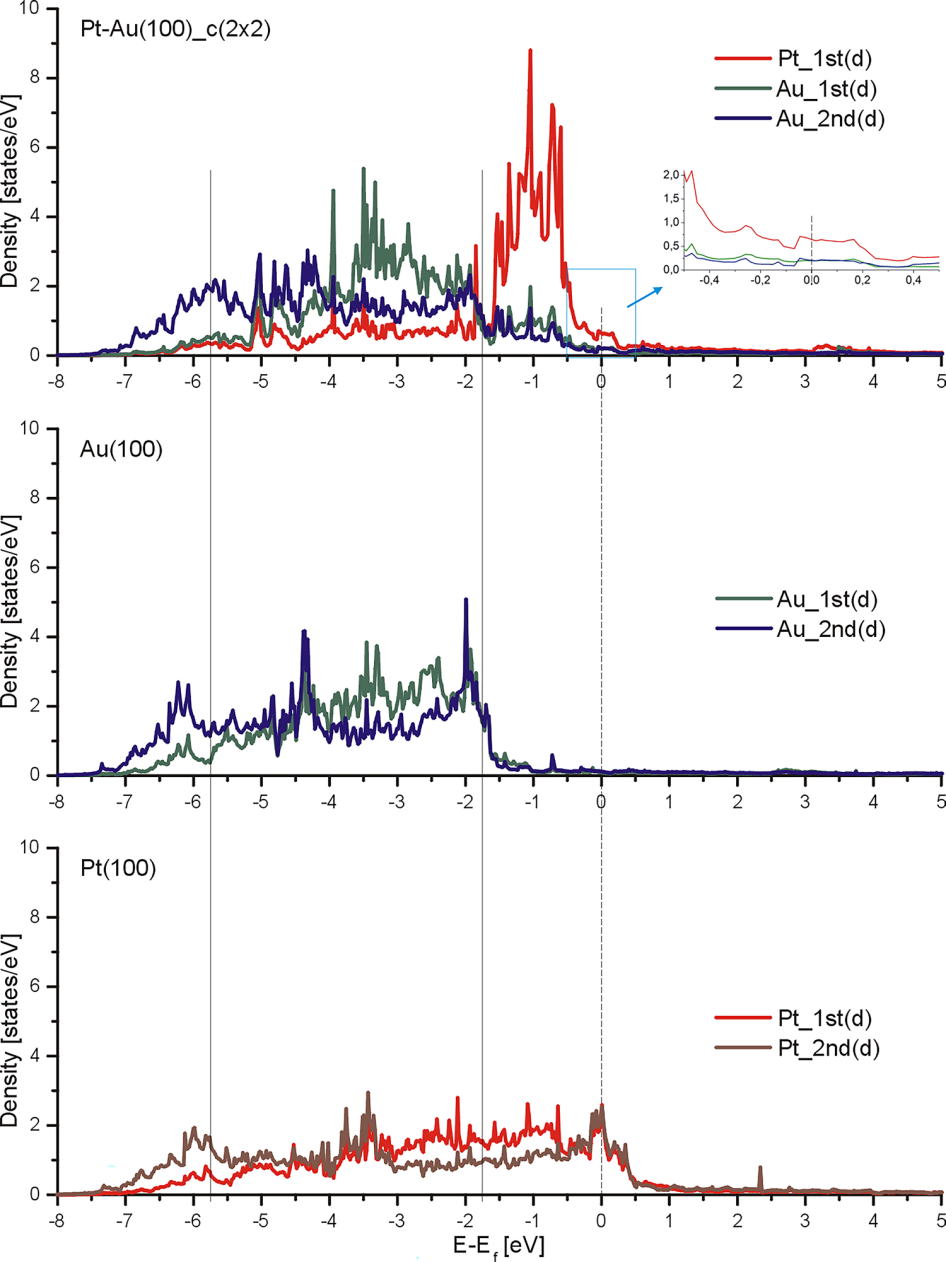
Local density of *d*-states obtained for Pt(100)-(1 × 1) (bottom image), Au(100)-(1 × 1) (middle image) and Pt/Au(100)-c(2 × 2) (upper image).

We will now discuss the influence of Pt atoms embedded into Au(100) topmost layer on the surface electronic structure. Figure 5 shows the local density of *d*-states near the Fermi level for 1st and 2nd atomic layers of Pt(100)-(1 × 1), Au(100)-(1 × 1), and Pt/Au(100)-c(2 × 2) phases. In the case of LDOS (*d*-states) for Au(100)-(1 × 1) two maxima can be distinguished at energies of 4.5 eV and 6.3 eV below the Fermi level. The energy positions of those maxima are in accord with previous ultra-violet photoelectron spectroscopy (UPS) investigations on the clean Au(100) surface where the characteristic *d*-band derived emissions at EBs of 4.6 eV and 6.4 eV were observed^31^.

It is visible for Pt/Au(100)-c(2 × 2) system that a strong increase of local density of states (LDOS) for platinum atoms at energy interval from −1.5 eV up to −0.5 eV occurs. Such states are not observed for Pt in the topmost layer of the Pt(100)-(1 × 1) surface. Our theoretical results are in line with the experimental data available in the literature^31^. Therein the additional feature centered at 1.0 eV below the Fermi energy for Au(100) after deposition of 1 up to 2 ML of Pt was observed by UPS. The structure was not detected for bare Au(100) surface as well as for thick layer of platinum and was ascribed to Au-Pt interface state^31^. Our calculations suggest that this additional feature results from the redistribution of Pt *d*-states in the Pt–Au alloy.

The LDOS of Pt/Au(100)-c(2 × 2) system for energy range + /− 0.25 eV around the Fermi level (see the insert) are almost constant for both Au and Pt atoms. Therefore the difference in the STM contrast between Pt and Au obtained for bias voltage in the range from around −0.25 V to + 0.25 V should be comparable, which is in agreement with the STM images presented in Fig. 4a,b.

**Figure 6:**
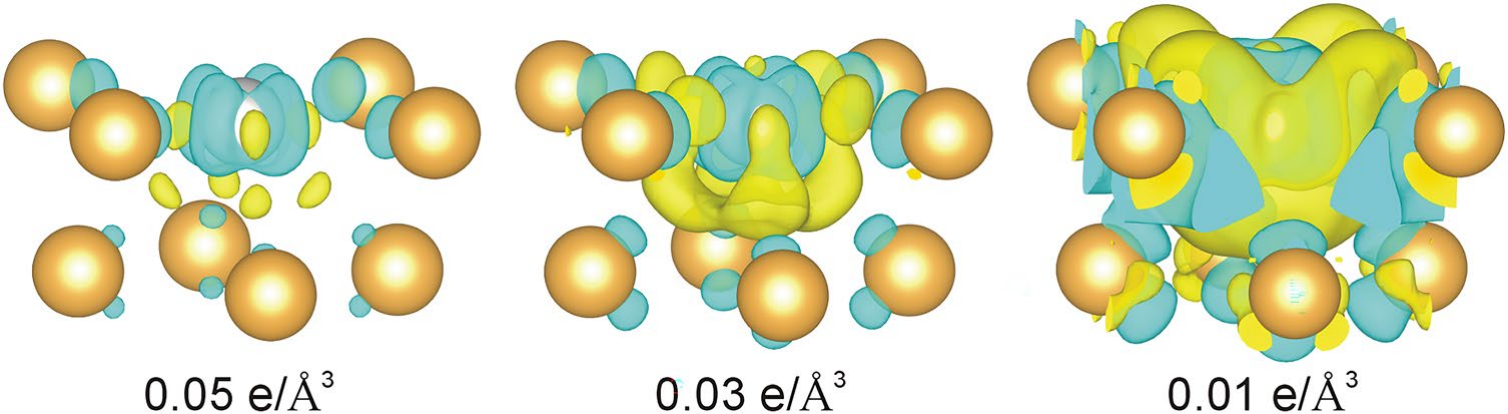
Isosurfaces of charge density difference for various levels. Yellow colour signifies accumulation and blue denotes depletion of electrons.

Further information about the system electronic properties is provided by the charge density differences (∆ρ) and Bader's analysis. The ∆ρ was calculated as ∆ρ = ρ^Pt–Au-c(2×2)^—(ρ^Au(100)_vacancy^ + ρ^Pt^), where ρ^Pt–Au-c(2×2)^, ρ^Au(100)_vacancy^ and ρ^Pt^ denotes the charge densities of the Pt/Au(100)-c(2 × 2) system, the bare Au(100)-(1 × 1) with vacancy in the place of Pt and the isolated Pt atom, respectively. Figure 6 presents isosurfaces of charge density difference for two topmost layers. As it can be seen, there is a depletion (blue) of electron density around the atoms. The charge flows from the atoms and accumulates (yellow) in the interatomic space forming a bond between Pt and Au. There is also a sphere of negative charge at the very top of the Pt atoms. Bader’s analysis reveals that there is a charge transfer into the first layer from the bulk (the second and deeper layers). Pt and Au atoms gain 0.11e and 0.06e, respectively. Thus the first layer of atoms is polarized, where Pt atoms are more negatively charged than Au. Compared to the bare A(100)-(1 × 1), this is an increase of 0.02e and 0.07e for Au and Pt respectively.

We will now discuss our results concerning the scenario of processes accompanying the Pt adsorption on Au(100) and the influence of Pt–Au alloy formation on the surface catalytic properties. The small rectangular islands, visible in the STM images for low Pt coverages (Fig. 1a,b), have monometallic composition and their shape and width are in accordance with those previously observed for Au islands on hex-Au(100)^19,28^. Moreover, they exhibit better thermal stability than bimetallic ones obtained for higher coverages. Indeed, after annealing at 100 °C for 10 min rectangular islands are still present on the surface, whereas the bimetallic dissolve. The bimetallic islands consist of flat phase and nanowire-like features. The latter have the same ‘magic width’ and atom arrangement as the small rectangular islands. They composition is bimetallic, however, gold is the major component. This allows us to conclude that the ‘precursors’ of nanowire-like features are small rectangular islands build of gold atoms. The growth process is comparable to one observed for Fe on Au(100)^25,26^. Firstly, the deposited Pt atoms exchange with gold ones which diffuse and create rectangular islands on the surface. Then, further depositions leads to exchange of atoms not only at the topmost layer of substrate but also at the islands—presence of bright species in the nanowire-like structures (indicated by arrows in Fig. 1d). Increasing the number of Pt atoms in nanowire-like feature may lead to atoms rearrangement into the (1 × 1) phase. The atoms creating the flat phase of islands come also from Pt adatoms deposited on the surface and Au atoms expelled from hex-stripes after lifting the substrate reconstruction.

As we have shown, the formation of Pt/Au(100)-c(2 × 2) alloy significantly influences the *d*-states of platinum, see Fig. 5. Above all, the surface gains the narrow *d*-band with the maximum centre around −1 eV. Therefore, we expect a stronger chemisorption and easier dissociation for molecules adsorbed on Pt/Au(100)-c(2 × 2) domains than on the Pt(100) surface. These electronic changes may contribute to the remarkable improvement of Pt catalytic properties in cyclohexene dehydrogenation to benzene for Pt/Au(100) system reported in Ref^29,30^.

## Conclusions

We presented the surface morphology evolution of hex reconstructed Au(100) influenced by the platinum deposition (up to 0.5 ML) at RT and then after annealing at 100–150 °C. At the beginning (below 0.2 ML) the presence of rectangular islands with hex-atom arrangement is found by STM. On the areas where the substrate surface is uncovered, the hex reconstruction of the topmost layer is preserved. Increase the amount of Pt, for range between 0.2 ML and 0.4 ML, leads to a change of the islands morphology. Now the coexistence of bimetallic (Au–Pt) nanowire-like features with flat phase of disordered Pt-Au alloy is detected. The sample annealing of Pt/Au(100) leads to dissolution of islands. The surface consists of the flat phase of Pt–Au alloy which coexists with the nanowire-like features. We showed that the number of atomic rows creating all nanowire-like features as well as the rectangular islands (observed for lower coverages) is quantized and in accord with the formula (6n + 1). After annealing there are small areas of Au-Pt flat phase which exhibit compositionally ordered c(2 × 2) structure. The DFT calculation of STM images of this structure reproduces the topographies of corresponding images provided by experiment very well and allows for exact atoms identification. Moreover the calculated LDOS reveal the strong redistribution of *d*-states for Pt embedded into Au(100)-(1 × 1) topmost layer in comparison to Pt(100)-(1 × 1). The enhancement of LDOS is observed for energy range between −1.5 and −0.5 eV. The charge density difference and Bader’s analysis reveal the bonding creation between Au and Pt atoms and charge transfer into the topmost layer, mainly Pt atoms. The changes of both structural and electronic properties of bimetallic Pt/Au(100) system with respect to monometallic ones, which were indicated by our present study, may significantly influence their catalytic properties.
